# Progress in Therapeutics

**Published:** 1874-06

**Authors:** J. H. Etheridge

**Affiliations:** Professor of Therapeutics and Materia Medica, Rush Medical College


ARTICLE III.—*Progress in Therapeutics.* By J. H. ETHERIDGE, Professor of Therapeutics and Materia Medica, Rush Medical College.

1. On the Specific Treatment of Typhoid Fever, after the Method of Dr. Brand, of Stettin. (*Lyon Medicale*, September, 1873.)

2. Typhoid Fever and Cold Baths. Prof. BEHIER. (*Practitioner*, Feb., 1874.)

3. Injection of Chloral in Tetanus. (*Medical Times and Gazette*, Feb. 28. From *L'Institut*, Feb. 18, 1874.)

4. Chloral and Camphor in Neuralgic Affections, Applied Locally. (*British Med. Journal.*)

5. Chloral in Labor. (*Lancet*, Feb., 1874.)

6. A New Use of Chloral. (*Annale de Chemicha*, 1874.)

7. A New Expedient in Administering Chloroform. (By JACOB HEIBERG, Secretary of the Norwegian Medical Society, Editor of "Norsk Magasin for Loege Videnskab," Christiana, Norway.)

8. Treatment of Diabetes Mellitus with Carbolic Acid. (*Berliner Klin. Woch.*, Dec. 8, 1873.)

9. Ammonia and Potassium Iodide.

1. The good results which the application of cold gives, in the treatment of typhoid fever, do not call for demonstration; but the opinion of physicians upon the favorable influence of this medication does not accord to it an absolute, *specific* value. Still, it is from this very point of view that M. Franz Glenard, interne of the hospitals of Lyons, pens an article, in which he gives us the method of Dr. Brand; one of the few German physicians, let us say parenthetically, who, during the war, interested themselves in the welfare of French soldier prisoners, and have merited, by so doing, an exceptional testimony of national recognition.

The following is what this method consists of. Its simplicity is only equaled by its constant efficacy:

As soon as the disease is known, the patient is put into a bath, the temperature of which is 20° centigrade, where he is put in *up to his neck.* The head is sprinkled with cold water of 6° or 8° (centigrade), affusion being indispensable in cases where there are cerebral phenomena. Affusion lasts for two or three minutes. For three or four minutes the patient is rubbed, shampooed, in water; then he is allowed to rest. In a few moments he experiences a violent shivering; the respiration is panting, and a cough supervenes; sometimes an involuntary stool takes place. The patient desires most urgently to leave the bath; he should be kept there at least fifteen minutes. Upon coming out he is shivering and purple, and presents "an aspect piteous enough to rend one's heart." His shirt is replaced without drying him, and he is put to bed, with a woolen blanket *over his feet;* the body is covered with a sheet in the summer and a light woolen blanket in winter. The patient is then given some lukewarm soup and a little old wine, and then he is left shivering, which may last from twenty minutes to an hour.

In three hours another bath; and so they are given, day and night, so long as the thermometer, placed in the rectum, does not mark more than 38.5°. After each bath, liquid alimentation, broth, milk and coffee, bread soup, *always tepid;* every quarter of an hour a swallow of ice water.

At the end of twenty-four hours the patient has taken eight baths. He is metamorphosed. The adynamia has disappeared, the cephalalgia is quieted, the tongue is moist. If he cough, the chest is enveloped in large cold compresses, renewed every quarter of an hour. The diarrhœa and the tympanites are likewise treated by means of cold compresses upon the belly. This amelioration is constant and does not cease. "It is not now necessary to reckon by the seven day periods to consider the symptoms, to calculate on the chances of a recovery. He will recover."

One of the inconveniences of this method, its greatest inconvenience (what method has not some?), is the exposing the patient, during the first few days, to a voracious, insatiable appetite, which the physician should guard against gratifying. He must struggle against the unreasonableness of his patient, "drying up his tears by kind words, correcting his faults, keeping a watch over his acts." As to other indications—there are none. As to complications—"there are none." "Cold water, more cold water *al*ways cold water; not a single medicament."

One hundred and seventy patients treated by Dr. Brand in 1868 gave 170 recoveries. Eighty-nine cases treated in Stettin in 1870–1871 furnished eighty-nine cures. However, the author admits that this leaves five cases of death out of his statistics, the patients having arrived from the twentieth to the thirtieth day after the accession of the fever. Brand has collocated 1411 cases treated according to his method by *various* physicians. The mortality is 4.7 per 100. Even this small mortality is not admitted by Brand, who avers, either that the patients were taken too late (after the first week) for treatment, or that his method was not rigorously enough used.

The pith of this method is summed up in this aphorism: *Every typhoid fever, treated regularly and from the beginning by cold water, will be free from complications and will be cured*—an aphorism that the physician will always bear in mind, and which "ought to be inscribed in letters of gold over the entrance to the bath room."

M. Franz Glenard denies vigorously that it is from enthusiasm, or that enthusiasm exists at all, in advocating this treatment; and that it well deserves its title, "*Specific Treatment*"—that is to say, a treatment which prevents the development of the disease, and uniformly cures it when it is developed. (Nysten.)

Far be it from us to challenge, in view of a confidence in it, almost impetuous, the incontestable value of treatment by cold water; but we would like to see its valuable effects on a larger scale. To start with, Brand's hypothesis is more than contestable. According to his idea, it is a fermentation wholly analogous to that of the barley germ. "The barley is the blood—the yeast the typh poison. On the one hand, elevation of temperature; on the other, the fever; the alcohol is the typh product." Fermentation lasts three days; typhoid fever three weeks. In lessening the temperature you cut short the fermentation; in cooling the patient you arrest the typh fermentation.

"I am right," adds the author, "in announcing that the advocacy of this treatment would bring to broad daylight the part played by hydrotherapy in typhoid fever, its essence—its right to be."

We must take the good as we find it. It is quite possible—very probable—that the hydrotherapeutic medication employed with this rigor (certain cases have taken more than 200 baths) can triumph over extremely grave cases; but all typhoid fevers do not respond so happily to a medication so difficult to initiate, and especially, to make acceptable to both the patient and family. It seems, according to the author, that up to the present time the physician has been disarmed in the face of a typhoid fever.

"Cold water is the only remedy to use—the only efficacy." Of course typhoid fever has its contingencies—its surprises (aside from the treatment of Brand, naturally)—but in the majority of cases the experienced physician can, toward the end of the seventh day, judge approximately of the gravity of the disease, and he distrusts a little, in spite of himself, the enormous statistics uniting in one immense sum total of facts naturally very dissimilar. The same treatment necessarily benefits the lighter cases, and the errors of diagnosis so frequent in the first eight days. Once more, we should not recoil before the treatment of Brand in a grave case, but it should be repugnant to us to generalize it, and we should the rather desire, in honor of the method, that this singular theory of fermentation and of typh alcohol should be suppressed in the beginning.

2. The February No. of *The Practitioner*, 1874, contains a translation from the *Bulletin de Therapeutique* of a clinical lecture by Professor Behier, delivered in the Hotel Dieu, in January of this year, on the subject of typhoid fever and cold baths.

He agrees with Brand in recognizing the unquestionable usefulness of these baths, but does not agree with him as to the cause of the excessive heat which the bath reduces. Brand regards heat as the result of *fermentation*, and rather arbitrarily—unwarrantably, perhaps—insists on the profession accepting this theory. Behier claims that the excessive heat may arise from three causes, and inclines toward accepting the last of the three. 1st, A diminution of the waste of heat, production remaining the same; 2nd, An increased production of heat, the waste remaining the same; and 3rd, A combination of the two preceding—*i. e.*, "an increase of combustion and a retention of caloric."

He recommends only three baths a day, of a temperature of about 65° F. Diarrhœa, albuminuria, heart disease, pulmonary and cerebral complications, pregnancy and collapse, are *not* contra indications to giving them. Wunderlich stipulates for a little warmer and less prolonged baths in cases complicated with heart disease or pregnancy. Tiemssen puts such patients into a warm bath, "the temperature of which is gradually lowered by adding cold water."

Prof. Behier says further: "We see, then, that if cold water moderates fevers, it is not perhaps by moderating combustions (for it is proved, on the contrary, that it tends rather to increase them), but by hastening the departure, the waste, of caloric. Its action is thus diametrically opposed to that of alcohol, quinine and other agents which directly attack and moderate the febrile combustion; its role is that of spoliation, not of economy of waste."

3. "M. Bouillard, on the part of M. Ore, a professor of the Bordeaux Medical School, related a case to the Academie des Sciences, February 16, in which chloral had been injected into the veins, as a remedy in traumatic tetanus. A man, after a wound of his finger, became the subject of tetanus, in consequence of which his mouth became so closed that no remedy could be administered. M. Ore therefore threw an injection, containing ten grammes (about 154 grs.) of chloral, into the veins, which produced peaceful sleep; and this was followed by a second and third injection, with the effect of obtaining a sleep of eight hours."

4. Chloral and camphor, equal parts, will yield, after a few hours standing, a clear fluid. This fluid is painted lightly over the painful part, and allowed to dry. It produces no irritation beyond a tingling sensation. It seems to be equally efficacious in neuralgia of any nerve, whether of the fifth pair or sciatic. Mr. Lenox Browne (*British Medical Journal*, March 7, 1874) recommends this combination very highly. He says that in every case it afforded *great*, and in some cases instantaneous, relief. He says he has "found it of the greatest service in neuralgia of the larynx, and in relieving spasmodic cough of a nervous or hysterical character."

5. The efficiency of chloral in *certain cases* of labor is astonishing. Prof. Playfair, of King's College, London, in the Feb. *Lancet*, says: "There is a type of labor very common, especially in women of a highly developed nervous organization, such as constitute a large proportion of our patients, among the higher classes, in which I have found chloral especially valuable. In these, before the rupture of the membranes, and the complete dilatation of the cervix, the pains are very severe, but short and ineffective, chiefly limited to the back, and producing little or no effect in dilating the os. Hours and hours of really intense agony often elapse, until the patient is wearied and exhausted by her fruitless sufferings. In cases such as these, a common and very useful practice has been to administer a considerable opiate, so as to produce some hours of refreshing sleep, after which we expect the labor to recommence with fresh vigor and effect. The disadvantage of this plan, however, is that during the action of the remedy the labor is suspended, and much time is thus lost. If, however, chloral is administered instead of the opiate, the probabilities are that the same refreshing rest will be obtained without any suspension of the pains or protraction of labor. The character of the uterine contractions will be observed to alter; they will become steady and useful, but they are not suspended."

Rigidity and spasm of the cervix, accompanied by tenderness upon digital examination, can be treated with the most marked success by using this drug.

6. Chloral possesses decided coagulating power on the blood. This property was first observed in 1870 by Prof. Luigi Porta. Since that time he has experimented in various ways with this drug, and has reached the conclusion that we have a new remedy for the radical cure of varix. By means of hypodermic injections he has treated and cured fifteen cases of varix of the leg. "He began by injecting 15 grs., but gradually reduced the quantity to 7, and ultimately to 5 grs., and injected this quantity * * * into numerous points, for the action of the remedy is greatest when it is injected at many separate points. Each of these injections forms a coagulum, and the patient should remain at rest for a few days to prevent slight phlebitis. The coagula undergo absorption, and the veins atrophy, or if they remain potent they cease to be varicose."

All accidents occurring from this use of chloral "have been very trifling." It is to be presumed that this mode of treatment is not limited to varices, but might be applied to other affections of the veins, such as varicocele. Prof. Porta reports one case of cure of this disease by injection. Prof. Coletti remarks: "That for some months in the wards of the Milan Hopsital, varicose ulcers have been successfully treated by the application of a solution of chlora hydrate, containing one part to four of water; recovery took place with great rapidity." (*Annale di Chemicha*, 1874, p. 353.)

7. Dr. Heiberg gives an account, in the *London Medical Times and Gazette*, Jan. 10, 1874, of a method he has resorted to for over two and a half years, for the avoidance of some of the inconveniences attending chloroform administration.

These inconveniences are, "incomplete, rattling respiration, pale, livid color of the face, feeble pulse, etc." The most distressing of these is the oppressed breathing.

His new expedient is "the drawing forward the lower jaw *in toto*." As soon as the rattling, incomplete respiration begins, he accomplishes the matter thus: "Standing, preferably, behind the reclining patient, the operator places both thumbs on the symphysis of the lower jaw, presses the second joint of the bent forefingers behind the posterior margin of the rami ascendentes of the under jaw, and thus holding the whole bone fast between the two hands, draws it forcibly forward (anatomically speaking). The most successful impulse is that which would be given if the intention were to lift the whole head and body by this grasp."

When the expedient is successfully performed—which is especially easy in children—a deep, complete respiration will immediately take place, and will continue as long as the jaw is "luxated" (*sit venia verbo*) forward. The obstacle to respiration is removed, the rattling ceases, and exactly the same result is obtained as if the tongue had been drawn forward, but with none of the inconveniences which would attend the latter. In over 1000 cases in which chloroform was administered, this has been the only expedient used, and it has always answered his purpose.

8. Drs. W. Ebstein and Julius Mueller, of Breslau, have published a few observations, in the *Berliner Klin. Woch.*, Dec. 8, 1873, on carbolic acid, in treating diabetes, which, though too few to base an opinion on, are valuable as being *suggestions*.

According to Dr. Pavy, diabetic sugar arises from fermentation, which turns amyloid substance in the liver into sugar. Carbolic acid arrests fermentation.

Drs. Ebstein and Mueller said: "Now we have it; give carbolic acid, check fermentation, and sugar will disappear." See the results on two patients:

The first was a strong man—or he had been till six weeks previous to seeking relief—aged forty-six years. Passed eight pints urine daily; specific gravity, 1032; amount of sugar, 2.86 per cent. He had lost thirty-four pounds in six weeks. Sexual power much diminished.

About five grs. crystal carbolic acid in peppermint water were taken daily. At the end of six days of taking this drug the sugar *disappeared* from the urine, and its specific gravity was 1013. His weight gradually increased from 173 to 185 pounds in the course of four months.

In the second patient, the same result followed. The acid was gradually increased till 7½ grs. were given daily.

Details of a third case are given, but the acid failed utterly in causing the sugar to disappear.

Bromide of potassium does not always succeed in epilepsy, yet we almost always try it. If carbolic acid cure or relieve a small percentage of cases of diabetes, we certainly owe much to Drs. Ebstein and Mueller for giving us the details of their cases.

9. Sir James Page first called attention to the fact that carbonate of ammonia greatly increases the therapeutic action of iodide of potassium. Five grains of the iodide of potassium, combined with three of ammonia carbonate, are equal to eight grains of potash salt administered in the ordinary way.

Such a combination is especially good in syphilis.

It is also highly recommended in internal aneurism—for relieving pain and assisting to consolidate the tumor.

---

*Electricity.* (The following translation is made from the "Journal de l'Anatomie et de la Physiologie," Nro. 2, 1874. The author is Dr. Onimus. Ed.)

"On the Difference of Physiological Action of Induced Currents, Acccording to the Nature of the Metallic Wire Composing the Helix."

We have investigated, from a physical, and especially from a physiological, point of view, the differences which the nature of the wire composing the inducing helices can cause.

We had inducing helices made in absolutely identical conditions: with wires of copper, with wires of lead, and with wires of silver.

The diameter of the wire was the same, and the length of these wires was 210 metres each (about 600 feet).

All the helices were constructed in the same manner, and were influenced in an identical manner by the inducing current.

Upon the nerves and upon the muscles of a healthy man the effects of a shock are different, according to the nature of the metal; and we might say, in a general way, that when the wire of the inducing helix is made of a metal that is a poor conductor of electricity, the contraction is stronger, and the impression upon the cutaneous nerves less lively than with wire which is a good conductor, as copper, for example.

The effects are as much more marked as the external resistance is greater. Thus, in making a current traverse alcoholized water, and in diminishing it to a minimum, so that muscular contractions scarcely occur, with the current from a helix of copper wire, we obtain *more* contractions, with the *same* conditions, with a current proceeding from a helix of silver wire.

Upon the superficial muscles, the difference between the currents from a helix of copper and those from a helix of silver is much less pronounced; it (the difference) shows itself in proportion to the thickness of the skin or the depth of the muscles.

The impression produced by the current on the lead wire, or on silver wire, is less intense; it extends less far upon the superficial nerves of the skin.

Upon the sensitive nerves, situated in the depth of the tissues, the excitation is perhaps more marked than that produced by a current on copper wire, but it is less acute and less lancinating.

We have completed these researches with the assistance of our regretted friend, Dr. C. Legras, in taking upon animals the tracing of muscular contractions produced by currents from the different helices.

The traces thus obtained indicate, in a very marked manner, the more energetic action of the current from the helix of silver.

In using a minimum current, and in experimenting in identical conditions, the curve which is formed by each muscular contraction is much more elevated by the silver helix than by the copper helix.

Further, with the silver helix, the contractions are regular and equal among themselves, and present, also, the double shock due to the current of the closing, and to that of the opening (of the circuit).

(The lever of the metronome was used as an interrupter. The text here contains five wood cuts illustrative of the various curves representing muscular contractions.—Ed.)

The tracing obtained from the silver helix shows the most irregular contractions, for many of them are faintly pronounced, and it is rare that they have the double shock (shown.) These differences are so much the more marked as one operates farther from the muscle and through the skin.

If the rheophores be thrust into the muscle whose tracing is registered, the difference still exists, but it is much more feeble. In this case, the trace which represents the contraction from the copper helix shows equally well the two shocks. (Figures 6 and 7 are here introduced, showing the different tracings for silver and copper respectively, which tracings differ materially.—Ed.)

In studying these traces, one recognizes, furthermore, that the shock produced from the lead or silver wire has a duration a little longer than that produced by the current from the copper wire.

These experiments prove that the tension in these conditions is stronger from the induced currents of lead or silver wire, and we have, at the same time, to remark that these differences, from a physiological point of view, resemble those which exist between the extra current and the induced current before mentioned.

If the currents from the lead or silver wire have, in these conditions, a tension greater than the current from a copper wire, they have, on the contrary, an inferior quantity, owing to the bad conductivity of these metals. Thus, in experimenting with these same helices, while the current supplied from the copper helix produced upon the galvanometer a deviation of twenty to twenty-five degrees, the current supplied from the lead or silver helix produced upon the same galvanometer a deviation of only one degree to a degree and a half.

If we recur to the tracings given above, we will see that they show in an incontestable manner that the tension has a manifest action upon the energy of the muscular contractions, and that when the excitation is directed upon the nerves and through the skin, the contraction is as much stronger as the tension is greater.

That which proves still more this influence of the tension, is this:

If we increase the length of the copper wire, so that the resistance will be the same from the copper helix as from the silver helix, the physiological effects are the same for the two helices; at least according to the evidence of the contractions, for the cutaneous sensibility is always more lively when excited by the currents furnished by the copper helix.

We easily see from these tracings, that when we operate directly upon the muscles the difference is much less pronounced, for the tension plays a part much less important. It is, verily, in this case, more necessary to have a penetration more or less deep, and the excitation, whatever it may be, suffices to provoke a contraction.

The sensations produced upon the skin also vary according to the nature of the wire, and the facts which we have observed prove, in addition, that this sensation is often independent of the tension, since a current having a stronger tension provokes a less painful impression than another whose tension is weaker but whose quantity is more considerable.

Finally, the rapidity of excitation should also be considered; and one can say, in a general manner, that the impression is vigorous in proportion as the current is of short duration.

All these facts show that the differences of physiological action between induced currents depend wholly upon the physical differences which exist in the production of these currents.

We have to remark, in closing, that, in the electro-magnetic apparati employed in medicine, it would be more advantageous to make use of silver wire rather than copper wire, for silver wire, of the same length, produces currents which penetrate more " deeply the muscles and produce less painful impressions upon the cutaneous surface. The induced currents from copper helices are only preferable in cases where we wish to produce a strong revulsion and a vigorous excitation upon the cutaneous nerves."

